# Clinical and engagement efficacy of a virtual musculoskeletal integrated practice unit in health system employees: aggregate and comparative results from two employers

**DOI:** 10.3389/fresc.2025.1541508

**Published:** 2025-11-05

**Authors:** Austin G. Cross, Usmaan Zunnu Rain, Eric C. Makhni, Emily N. Schnettler, Prakash Jayakumar, Sameer Berry, Jeff Vandenboom, Courtland Keteyian, Russell Day, Daniel Verhagen, Michael Schumacher, Joseph Kucksdorf, Bruce Muma

**Affiliations:** 1 Texas Tech University Health Sciences Center, Lubbock, TX, United States; 2 Henry Ford Health, Detroit, MI, United States; 3 Michigan State University, East Lansing, MI, United States; 4 Massachusetts Institute of Technology, Cambridge, MA, United States; 5 University of Michigan, Ann Arbor, MI, United States; 6 Emplify Health, Green Bay, WI, United States; 7 Wayne State University School of Medicine, Detroit, MI, United States

**Keywords:** virtual care, patient outcomes, integrated musculoskeletal disease management, integrated practice unit, employee health

## Abstract

**Introduction:**

Integrated Practice Units are whole-person models of care designed to deliver a comprehensive range of treatment strategies centered around a patient's preferences, values, and needs. The purpose of this study was to assess the efficacy of a virtual IPU (V-IPU) for employees of two large health systems experiencing back, neck or joint pain. Specifically, we evaluated improvements in pain interference, physical health, and user satisfaction/experience.

**Methods:**

This was a prospective cohort study with a total of 167 employees from two health systems who were recruited through e-mail outreach and completed a brief health assessment, including patient reported outcome measures (PROMs) for physical, emotional, and pain health. Upon sign-up, employees began a 12-week multidisciplinary program consisting of musculoskeletal (MSK) physician telehealth treatment and oversight, supervised one-to-one physical therapy, registered dietitian counseling, health coaching, and platform to in-person specialty services when clinically appropriate. National Institutes of Health (NIH) Patient-Reported Outcomes Measurement Information System (PROMIS) scores for physical health, mental health, and pain interference were assessed at intake, 6-weeks, and 12-weeks after program initiation. Net promoter score (NPS) was measured to evaluate participant experience and satisfaction with the program.

**Results:**

The average age was 50.56 years, and a large majority of responders were female (89.2%). There were clinically meaningful improvements for PROMIS measures of physical health, mental health and pain interference (5.6, 4.4 and 6.9 points, respectively). The Net Promoter Score was 85 for engaged individuals. Additionally, the V-IPU was successful in connecting employees to additional surgical-avoiding services offered by the employer and which complemented the digital participation of the V-IPU.

**Conclusion:**

The V-IPU improves health outcomes and care coordination for health system employees. These findings support the use of virtual multidisciplinary models to enhance access and outcomes in employer-based health initiatives.

## Introduction

Musculoskeletal (MSK) conditions, such as those of the back, neck, and joints, continue to be among the most costly to self-insured employers, with regards to both lost productivity and costs of care (1) and despite well-intentioned investments through wellness benefits programs (2). These costs, primarily borne by employees and employers, are compounded by a general trend within US and global health care of consolidation and inflation-impacted costs of care (3). In 2016 alone, the cost of neck and back issues was $134.5 billion, which was the highest rate of spending for both employers and employees out of 154 conditions (4).

Traditional management of musculoskeletal conditions involves care delivery via fee-for-service pathways prioritizing quantity over quality and volume over evidence-based treatment strategies providing improved health outcomes, tailored to the patient's needs. Recent studies demonstrate significant over-utilization of costly interventions (including those that lack sufficient evidence, such as the use of biologic injections, advanced imaging, and arthroscopic washout of the knee for osteoarthritis) within fee-for-service settings (5). Studies estimate up to 30% of joint replacement (6) and 50% of back/spine (7) surgeries may be performed inappropriately leading to sub-optimal outcomes, experience, and failure to meet patient expectations.

One strategy to combat this overutilization is the integrated practice unit (IPU), as described by Porter and Lee (8). There have been a growing number of IPUs developed across medical specialties including those designed for longitudinal condition-based management of musculoskeletal conditions. Musculoskeletal IPUs have demonstrated value through improved health outcomes benefiting patients at lower cost (9). Compared to traditional fee-for-service care, IPUs are differentiated by rigorous measurement and assessment of patient reported outcome measures (PROMs)—which are patient-centered assessments of core health domains including capability (valued life activities), symptom intensity, cost measurement and psychosocial health—and multidisciplinary management of the disease/condition. Unfortunately, very few IPUs have been implemented due to logistical and administrative barriers in their delivery.

These challenges have provided an opportunity for leveraging digital health (telehealth and remote care delivery solutions) to enhance multidisciplinary team-based care, appropriate treatment selection, data-driven decision support and care coordination that exemplifies the multi-faceted benefits of IPU-based care. Further, there is a critical need for integrating such digitally enabled care with in-person services to ensure cohesive transition from virtual care to local, in-person specialty non-surgical and surgical care. While many digital solutions focused on exercise therapy are currently on the market, most rely on technology-lead, asynchronous care, with limited clinician oversight or interaction. There is a scarcity of solutions led by clinical care teams of physicians and physical therapists providing real-time care and counseling as is performed in an IPU.

We sought to understand the impact of a Virtual-first IPU (V-IPU; Protera Health, Troy, MI) focused on providing whole-person, multidisciplinary, musculoskeletal care in an employee population of two large self-insured health systems. Our primary objective was to assess change in PROM scores measuring capability (valued life activities) and symptom intensity. Secondarily, we assessed level of improvement in mental health, patient activation (defined as the knowledge, skills, and confidence in engaging in one's health and health care), and patient experience. Finally, we assessed process-level measures including engagement frequency, level of care coordination related to in-person health services and appointments. Positive findings could justify use of a V-IPU model for other self-insured employer populations around the country.

## Methods

This was a prospective cohort study with IRB approval, and was conducted at two health systems, Health System A (Henry Ford Health System, Detroit, MI) and Health System B (Bellin Health, Green Bay, WI). Employees with active symptoms of back, neck, or joint pain self-enrolled into the V-IPU in response to email awareness of the program offering. The email contained a link to the program sign up page and intake health assessment (including PROMs). Employees were recruited into the study through an e-mail posting by the employer, with a link to the program sign up page and intake health assessment (including PROMs). Employees agreeing to experience the V-IPU completed an intake survey that collected employee information such as demographics, habits, and condition, as well as baseline PROM scores and other health measures, such as anxiety and depression symptoms. After completing the intake survey, the employee was contacted by phone and scheduled for a virtual enrollment visit with the care team from the V-IPU. This care team consisted of a licensed MSK physician specialist, doctor of physical therapy (DPT), registered dietitian, and health coach. Each employee underwent a series of telehealth counseling appointments with the care team providers in an individualized program based on the employee's condition and symptom severity. The desired outcome was for the employee to complete a digitally-based exercise program while gaining disease education. Moreover, the educational content delivered were intended to improve the employees' “activation”, consisting of understanding, skills, and confidence in making positive lifestyle decisions (i.e., around tobacco use, opioid use, nutrition, mental health, and other wellness habits). The employee underwent a check-in every six weeks to ensure that PROMs were improving, and the patient was satisfied. Adjustments were made accordingly.

The V-IPU provided highly coordinated care involving a range of treatment strategies centered on PROMs. These quality metrics provide quantitative reporting about patient health across numerous domains, such as physical function, pain interference (impact of pain on quality of life), and mood. In an effort to standardize the care delivery, a single care team was assigned to this employee population. As part of the V-IPU, PROMs are collected at baseline, mid-program (approximately 6 weeks), and conclusion of the program (approximately 12 weeks) (Table 1). The care program is individualized according to PROMs in numerous ways:

- Treatment selection: patients with PROM scores in “moderate” or “severe” score ranges received accordingly more intensive oversight and participation by a musculoskeletal physician, in addition to the standard of care treatment delivered by the V-IPU care team (physical therapy, nutrition counseling, health coaching).
- Predictive care modeling: in accordance with current literature (10, 11)^,^ patients with PROM scores in the normal mild ranges for function and pain were selectively treated with the non-operative model through the V-IPU, while those in the moderate and severe ranges were more readily connected to in-person specialty care for surgical evaluation if not showing early improvement with the non-operative program. For example, a patient with known advanced osteoarthritis of the knee and “severe” domain scores for pain and/or function would be more readily referred to a partner (in-person) orthopedic surgeon from the V-IPU platform, as the likelihood of success following surgical treatment would be higher compared to patients with similar degree of osteoarthritis but with normal or mild PROM domain scores (12).
- Educational programming: Certain PROM domains were used to individualize educational programming. Those with positive screens on anxiety screens (GAD-2) or moderate/severe findings for pain interference were provided educational programming that focused on pain coping techniques. This content was recorded by licensed pain psychologists and integrated with the patient's application experience.
- Activation and Engagement: to improve lifestyle-modification and self-care techniques with patients, the V-IPU assessed baseline activation scores to identify those with low activation who would benefit from more intense oversight by the V-IPU care team, as opposed to those with high baseline scores who would be well-served through a lower-touch approach. Activation was assessed through a de-novo assessment tool, and those scoring less than 15 points were provided with more frequent touchpoints to ensure compliance with and ability to extract practical knowledge from educational videos including personalized exercises, educational resources within the 12 week program.

**Table 1:** Variables defined.

Domain	Measure	Description
Physical Health	NIH PROMIS Global-10 (PH component)	Measure of overall physical health
Pain	NIH PROMIS Pain Interference sF-4	Measure of impact of pain on quality of life
VAS Pain	NIH PROMIS Global-10	Pain rating (from 0 to 10) as reported from the PROMIS Global-10
Activation	Protera Activation Score	A novel measure of a patient's abilities, skills, and understanding in making positive lifestyle decisions
Mental health	NIH PROMIS Global-10 (MH component)	Measure of overall mental health
Anxiety	GAD-2	Anxiety screen
Depression	PHQ-2	Depression screen

SOURCE Image credit; Dr. Eric Makhni.

A core principle of the V-IPU intervention was to improve participant self-guided exercise and education about his/her condition and exercise program. To achieve this, each participant received weekly “nudges” which consisted of reminders for exercise compliance, notification of new exercise assignments, and assignment/notification of educational content. Most of the education content was video-based and presented by multi-disciplinary specialists, such as physical therapists, sleep specialists, orthopedic surgeons, pain psychologists, registered dietitians, and obesity physicians. Each educational video content was approximately 2–4 min in length, and 1–2 different videos were assigned each week.

Participation in the program was designed to be approximately 3 months long. Once enrolled, the employee would receive weekly or biweekly supervised physical therapy encounters and be expected to complete home-based self-guided sessions through the web portal exercise program interface. PROMs and clinical outcomes were assessed at intake as well as at the midpoint (6-weeks) and conclusion of the engagement. Employees who experienced symptom improvement earlier and as evident by PROM scores were offered an early termination at the program midpoint if they felt that they had sufficiently improved. Employees who sustained injury exacerbation, acute injury, or any other concerning symptoms were escalated to in-person specialist through the health platform and with assistance of the v-IPU care team.

Several different PROMs and clinical outcomes were measured through the engagement and were collected electronically through the V-IPU online interface. These measures can be seen in Table 1. The core domains included physical health, emotional health, pain health, and activation (defined as the understanding, skills, and confidence needed for making positive lifestyle decisions). National Institutes of Health (NIH) Patient-Reported Outcomes Measurement Information System (PROMIS) (13) tools were used for many of these assessments. The threshold of 2.5 points was defined to be the minimum clinically important difference (MCID) (14, 15).

Additionally, the impact of the digital solution on in-network care coordination was also assessed. This included ability to steer employees toward in-person providers for orthopedic consultation as well as to guide employees to preferred in-person health benefits services. These services were prioritized by the employer due to the promotion of non-surgical treatment for back and joint pain and included massage therapy, chiropractor services, acupuncture, along with nutrition and lifestyle medicine services.

Finally, engagement and experience were measured throughout the intervention through electronic logs that tracked webportal usage for exercises and educational content. Engagement metrics included number of care team appointments attended, number of self-guided exercise days completed (as defined by any day in which an exercise was accessed by the participant), total active days on the web portal, number of educational videos accessed, and proportion of participants that viewed/accessed the in-network services. As this was a prospective cohort study, no comparative statistical analyses were performed. Any missing data secondary to attrition was not calculated in the data analysis in an intention-to-treat methodology.

## Results

One-hundred and sixty-seven employees completed the initial intake form and care team virtual visit (Age, Mean: 50.56, SD: 11.87 Female *n* = 149, 89.2%) (Table 2). The most common primary condition was back pain (*n* = 79, 47%). The average body mass index, or BMI, was 29.6, and 19.4% had a BMI > 35 (defined as Type-II obesity).

**Table 2:** Population demographics.

Demographical variable	Number of participants, (*N* = 167)	Percentage (%)
Age		
<50	68	40.7
50–60	57	34.1
61–70	42	25.1
71–90	0	0
Sex		
Male	18	10.8
Female	149	89.2
Race		
Black/African American	11	18
White	39	63.9
Hispanic	2	3.3
Asian	3	4.9
Other	0	0
Mixed	6	9.8
No response	106	
BMI		
<18.5	0	0
18.5–24.9	49	29.7
25–30	49	29.7
30–35	35	21.2
35–40	15	9.1
>40	17	10.3
No response	2	
Chief complaint		
Knee	13	7.8
Arm/Elbow	2	1.2
Foot/Ankle	7	4.2
Hand/Wrist	4	2.4
Hip/Thigh	15	9
Neck	26	16
Shoulder	14	8.4
Back	79	47
Other	7	4.2
Unknown	0	0

SOURCE Image credit; Dr. Eric Makhni.

**Figure 1:**
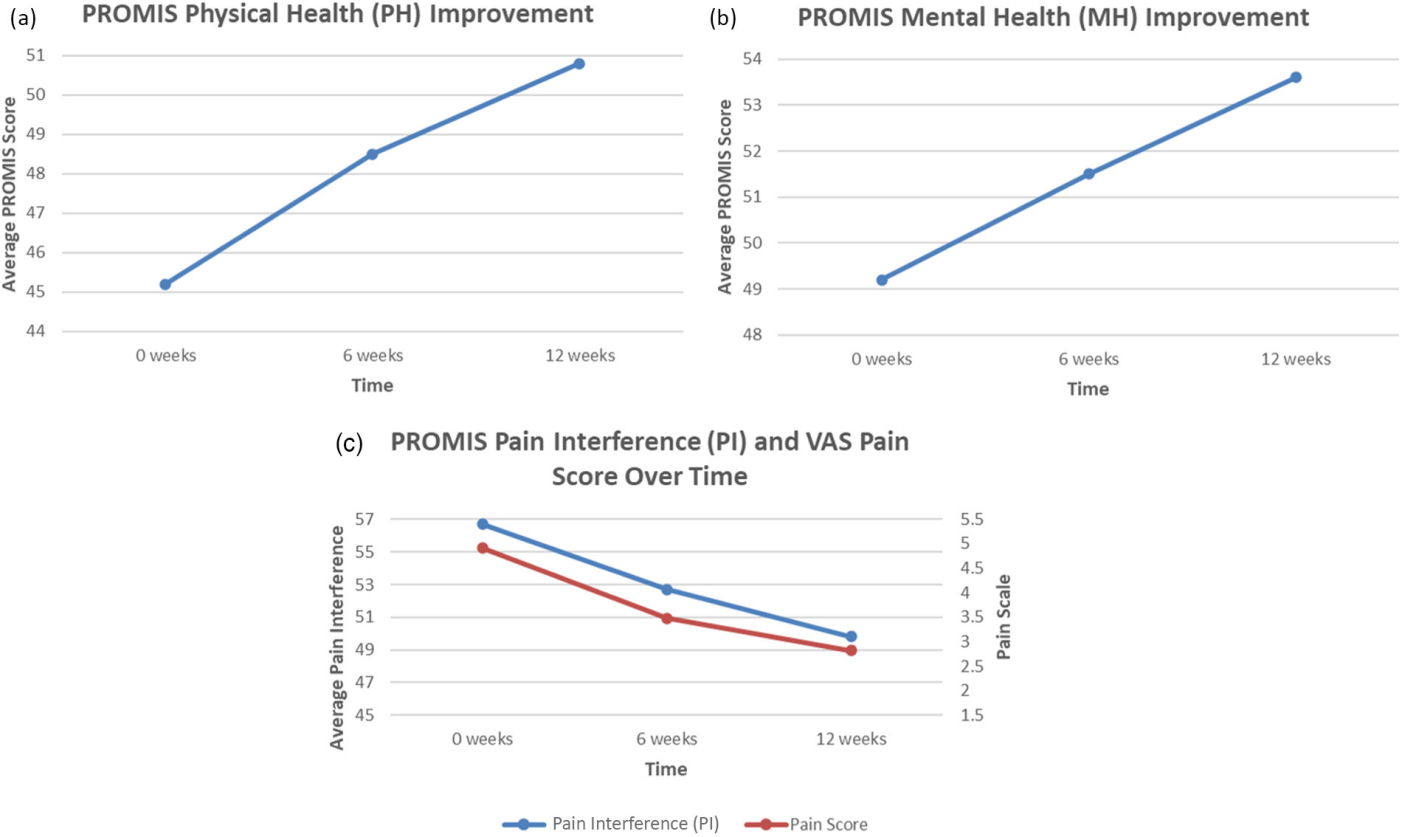
**(a)** Average change in PROMIS global-10 physical health scores for employee cohort over a 12-week period, which is shown to exceed the target clinical improvement of 47.4. **(b)** Average change in PROMIS Global-10 Mental Health scores for employee cohort over a 12-week period, which is shown to exceed the target clinical improvement of 51.5. **(c)** Average change in PROMIS Global-10 Pain Interference and VAS scores for employee cohort over a 12-week period, which is shown to exceed the target clinical improvement of 54.4. SOURCE Image credit; Dr. Eric Makhni.

**Figure 2:**
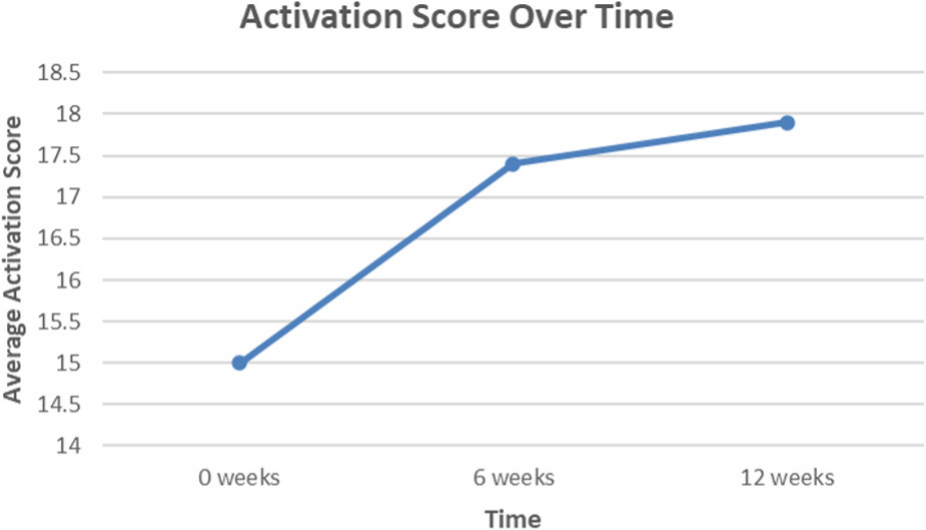
Average change in PHQ-2 (depression) and GAD-2 (anxiety) scores reported by employee cohort over a 12-week period. Black represents Patient Health Questionnaire-2 (PHQ-2) scores; Grey represents Generalized Anxiety Disorder-2 (GAD-2) scores. SOURCE Image credit; Dr. Eric Makhni.

**Figure 3:**
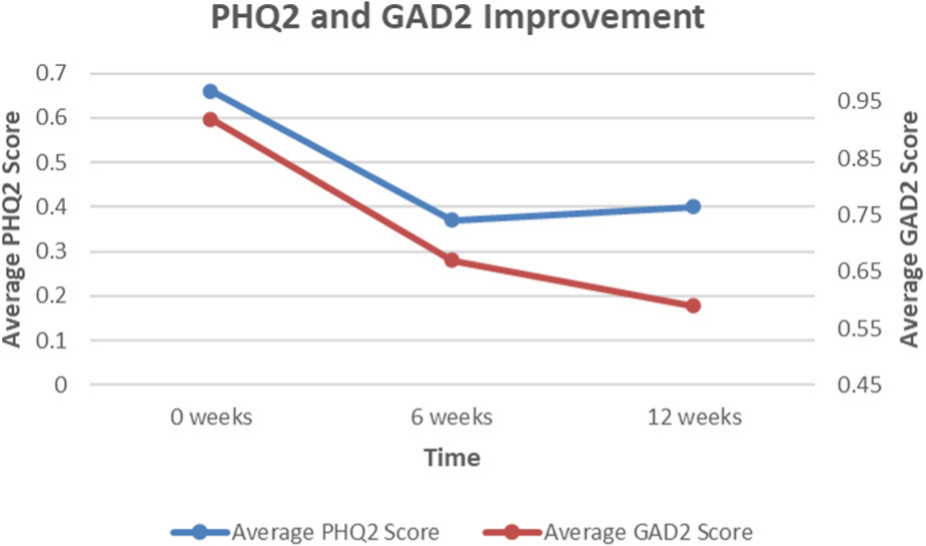
Average change in activation scores for employee cohort over a 12-week period, which is shown to exceed the target clinical improvement of 17.5. SOURCE Image credit; Dr. Eric Makhni.

Of the actively engaged employees that completed the program, there were significant improvements in all three domains of physical, mental, and pain health (Figures 1A–C). For physical health, the average NIH PROMIS Global-10 Physical Health component score change was 5.6, which exceeded (14) the threshold for minimum clinically important difference (MCID) of 2.5 points. For pain interference (impact of pain on an individual's quality of life) and mental health, this improvement was approximately 6.9 points and 4.4 points, respectively. For GAD-2 anxiety screen (Figure 2**)**, the average score improved from 0.92 to 0.59, which is a 35.7% score improvement. When considering the PHQ-2 depression screen, the average score improved from 0.66 to 0.40, which is a 40.0% score improvement. Finally, for patient activation (Figure 3**)**, there was an improvement from 15 to 17.91, denoting a 19.4% improvement.

When considering engagement (Table 3), metrics centered around utilization of the web portal and associated services. On average, of the cohort that completed the engagement, participants logged into the portal approximately 3.36 times per week, with an average of 43.67 logins total. Participants viewed an averaged 1.61 (per week) and 19.36 total pieces of educational material over the engagement period. The Net Promoter Score (a quantitative measure) of this cohort was 85, indicating very high levels of satisfaction with the program (16).

**Table 3:** Webportal engagement metrics.

Total population	*N*
	167
Measure	*Mean*
Webportal engagement	
Exercise material accessed	10.63
Total logins	43.67
Average logins per week	3.36
Education consumed	19.36
Education consumed per week	1.61

SOURCE Image credit; Dr. Eric Makhni.

As part of the engagement, multiple non-surgical services were made available and promoted to employees, including acupuncture, chiropractor, massage, and lifestyle management services. Additionally, if there was clinical concern, the participant was connected to in-person orthopedic physicians for specialty consultation.

## Discussion

Multidisciplinary team-based care for back, neck, and joint pain that provides a comprehensive range of non-operative strategies through a virtual format leads to improved health related outcomes, patient experience, patient activation, care coordination [and other utilization metrics], for employees experiencing a range of musculoskeletal conditions. These improvements signal opportunities for not only improving health and wellness for populations of employees but also substantial reductions in costs through highly coordinated care that averts patients from specialty care while enabling appropriate resource utilization.

Notably, there are a growing number of companies developing physical therapy via digital health platforms (15). While studies have demonstrated their cost-effectiveness alongside reductions in medical claims (18), few have evaluated efficacy utilizing a comprehensive range of PROMs, patient experience, patient activation, and metrics related to in-network care coordination. More importantly, physical therapy is only one of several treatment components of the V-IPU.

We utilized a wide range of validated PROMs as our principal metric of meaningful improvement, which unlike process level measures, provide a gauge of care quality based on the assessment of important health domains (19). Further, we utilized well-established thresholds for improvement applied to these validated instruments to define the level of impact. The National Institutes of Health (NIH) PROMIS assessment tools used in this study are fast becoming validated tools of choice that provide domain-level health assessment with increased precision and efficiency compared to legacy instruments (20). For instance, PROMIS Pain Interference, used as a pain measure, assesses the impact of pain on an individual's quality of life. Scores generated using this tool are substantially more meaningful and relevant to the impact of musculoskeletal disease on an individual in the context of their lived experience with their condition than traditional pain scoring using a “0–10” visual analog scale (VAS) (21). Ultimately, engagement with the V-IPU leads to clinically meaningful improvements across the domains of capability, symptom intensity, alongside mental health.

The V-IPU also demonstrates improved patient activation over the course of the 12-week program, which has also been linked to improved physical function in patients with back pain (22). The concept of activation encompasses aspects of resiliency, behavior change, and agency—taking responsibility for one's health. Our findings suggest the opportunity to improve agency within a relatively short period of time through the delivery of high-quality virtual care. While a modest number of studies have utilized patient activation measures in musculoskeletal research, to our knowledge, this is the first study to apply this concept in the context of virtual comprehensive care for patients with musculoskeletal conditions.

Our work also evaluates the ability of the platform to coordinate connections and transitions of care for employees participating in the program to in-person services. This component is lacking in several “plug-and-play” digital health solutions that aren't integrated into health systems and are thus limited in enabling a level of care transition to in-person health services required to truly deliver the most appropriate treatment at the appropriate time (23). Protera Health was designed to solve for this deficiency using a platform component that was accessible for participants and formed through collaboration with the employer, similar to previously reported programs in the literature (24). Our findings demonstrated both a successful ability of the platform to coordinate in-person care needs for participants to orthopedic specialty care, as well as bolster the utility of non-surgical, non-specialty care and avoiding surgery.

This study is not without limitations. The participant cohort was from two health system employers, and the results may not be generalizable to all employee populations. However, the health systems in this study encompasses a large regional geography with an ethnically and socioeconomically diverse population. Another limitation was the inability to correlate engagement with impact on total cost of care or orthopedic cost of care. Isolating musculoskeletal costs of care can be extremely challenging when considering the need for longitudinal assessment, comparison groups, and sophisticated claims-based analytics. There were additional limitations, including lack of a control group or ability to control for selection bias (i.e., enrollment of employees that were self-motivated to complete the program). Participant digital literacy, socioeconomic status and geography may influence patients ability to access a digital platform, however recent literature suggest that digital telehealth solutions can be effective in older-aged orthopaedic patients (25). While there was no correlation of PROMs to clinical outcomes (i.e., radiographs, surgical findings, etc.), the inclusion of PROMs allows for patient-centered ratings of health that is considered to be the most relevant outcomes available. Also, it should be noted that a separate analysis for patients lost to attrition or those who went onto surgical intervention were not studied. Finally, the study had a relatively small sample size and recruited from two employers, which may limit the generalizability of the study findings. Future research considerations would address these limitations.

In summary, a V-IPU can achieve improved health-related outcomes, patient experience, activation, and highly coordinated care between a virtual format with in-person health services. The use of PROMs and measures of patient activation are both important for decision support within this model alongside improvement in the quality of care delivered. Further evaluation, including comparative effectiveness studies, of this novel and comprehensive approach to common musculoskeletal conditions is required to evolve the model and scale it across different archetypes of the health system.

## Data Availability

The original contributions presented in the study are included in the article/Supplementary Material, further inquiries can be directed to the corresponding author.
